# Clinical Laboratory Terminology Standardization for Semantic Interoperability Using a Large Language Model–Based Agent: Methodological Study

**DOI:** 10.2196/92499

**Published:** 2026-08-20

**Authors:** Lijuan Wu, Jinxin Huang, Hongnian Wang, Liyi Mai, Xueyun Zhan, Xinrong He, Xiaotang Zhang, Huiying Liang, Xin Li, Abdelouahab Bellou

**Affiliations:** 1 Institute of Sciences in Emergency Medicine, Department of Emergency Medicine, Guangdong Provincial People’s Hospital (Guangdong Academy of Medical Sciences), Southern Medical University, No 106, Zhongshan Second Road, Yuexiu District, Guangzhou, 510080, China; 2 Medical Research Institute, Guangdong Provincial People’s Hospital (Guangdong Academy of Medical Sciences), Southern Medical University, Guangzhou, China; 3 Key Laboratory of Digital-Intelligent Disease Surveillance and Health Governance, North Sichuan Medical College, Nanchong, China; 4 Medical Big Data Center, Guangdong Provincial People’s Hospital (Guangdong Academy of Medical Sciences), Southern Medical University, Guangzhou, China; 5 Department of Emergency Medicine, Guangdong Provincial People’s Hospital (Guangdong Academy of Medical Sciences), Southern Medical University, Guangdong, China; 6 Key Special Project of Intergovernmental International Science and Technology Innovation Cooperation, China-Algeria Joint Laboratory on Emergency Medicine and Immunology, Guangzhou, China; 7 Department of Emergency Medicine, Wayne State University School of Medicine, Detroit, MI, United States; 8 Global Network on Emergency Medicine, Brookline, MA, United States

**Keywords:** large language models, laboratory test standardization, retrieval-augmented generation, semantic interoperability, Logical Observation Identifiers Names and Codes, LOINC mapping, agent-based systems

## Abstract

**Background:**

Semantic interoperability, the ability of disparate health information systems to exchange and consistently interpret clinical data, is a cornerstone of modern digital health, underpinning cross-institutional research, real-world evidence generation, and global health surveillance. Laboratory tests constitute one of the richest clinical data sources, yet multilingual variation and institution-specific naming conventions severely impede their standardized integration across systems.

**Objective:**

We propose LabBridge, a large language model (LLM)–based agentic framework designed to standardize laboratory tests to the Logical Observation Identifiers Names and Codes (LOINC) standard, enabling cross-lingual semantic interoperability while minimizing reliance on language-specific rules and manual curation.

**Methods:**

LabBridge integrates linguistic normalization, hybrid retrieval (combining domain-adapted embeddings with the LOINC ontology structure), and constrained LLM reasoning within an agentic workflow that enforces terminological consistency and traceability. We evaluated the framework on 2 real-world laboratory datasets from emergency department patients, one in Chinese and one in English, representing cross-lingual and cross-institutional heterogeneity. Performance was assessed across 5 LLMs and compared with vector-based baseline (BGE-M3, Beijing Academy of Artificial Intelligence) and retrieval-augmented generation (RAG) approaches, using mapping accuracy against a curated reference set of clinically relevant LOINC core codes as the primary metric.

**Results:**

At full coverage (Top@100%), LabBridge achieved 81% to 90% LOINC mapping accuracy across 5 LLMs on both Chinese and English datasets, outperforming all baseline methods (*P*<.01). On the Chinese dataset, it improved over BGE-M3 by 66 percentage points (90% vs 24%) and over the best RAG method by 41 percentage points (90% vs 49%). On the English dataset, gains ranged from +4 to +19 percentage points over RAG baselines. The framework maintained robust performance across frequency strata, including the relatively lower-frequency stratum within the selected evaluation datasets. Notably, on the Guangdong Provincial People’s Hospital (GDPH) dataset, LabBridge achieved 85% accuracy on high-frequency terms (Top@30%) compared to 93% on low-frequency terms (Bottom@30%). The highest accuracy, 90% in both the languages, was achieved using DeepSeek-V3 (Hangzhou DeepSeek Artificial Intelligence Co, Ltd), with GPT-4o (OpenAI; 88%‐89%) and GPT-4o-mini (OpenAI; 87%‐90%) showing comparable results.

**Conclusions:**

LabBridge demonstrates that embedding LLMs with an ontology-aware, agent-coordinated architecture enables effective standardization of laboratory data. By unifying semantic retrieval, linguistic normalization, and constrained reasoning, the framework accelerates the terminology standardization process by transforming expert effort from manual code lookup to candidate verification. These findings offer a practical pathway toward scalable, auditable semantic interoperability in health care ecosystems.

## Introduction

Semantic interoperability, the ability of disparate health information systems to exchange, interpret, and use clinical data consistently, is a cornerstone of modern digital health [1-3]. It underpins critical applications such as cross-institutional cohort identification, real-world evidence generation, public health surveillance, and global clinical trial recruitment [4-6]. Despite decades of standardization efforts, however, achieving true semantic interoperability remains elusive, particularly in the domain of laboratory medicine [7,8]. Laboratory test results constitute one of the richest and most frequently used data types in clinical care, yet their representation varies widely across languages, institutions, and electronic health record systems [9]. Local naming conventions, abbreviations, and language-specific phrasing often obscure the underlying clinical meaning, rendering automated data integration error-prone or infeasible [10,11].

To address this challenge, controlled terminologies such as Logical Observation Identifiers Names and Codes (LOINC) have been developed to provide a universal lexicon for laboratory and clinical observations [3,12]. While LOINC adoption has grown globally, its practical implementation is hindered by the labor-intensive process of mapping local test descriptions to standardized codes, especially in multilingual settings [13]. Traditional approaches rely on manual curation, rule-based matching [14], or simple lexical similarity [15], which scale poorly across languages and fail to capture contextual or synonymous variations. More recent methods leverage vector embeddings or retrieval-augmented generation (RAG) to improve mapping accuracy [16], but they often treat standardization as a passive retrieval task, lacking mechanisms to enforce terminological consistency, resolve ambiguity, or provide auditable reasoning—critical requirements in regulated health care environments [17].

Large language models (LLMs) offer new opportunities for clinical natural language processing, with demonstrated capabilities in understanding and generating medical text [18,19]. However, naively prompting LLMs to perform terminology mapping frequently yields invalid or hallucinated codes due to the absence of explicit constraints from domain ontologies [20]. Moreover, most existing LLM-based solutions are evaluated only in monolingual, high-resource contexts (eg, English), leaving a significant gap in support for low-resource or non-English languages that dominate much of the global health landscape [21,22].

To bridge these gaps, we propose LabBridge, an agentic LLM framework specifically designed for ontology-grounded standardization of laboratory test descriptions to LOINC. Unlike prior work, LabBridge coordinates multiple stages, such as linguistic normalization, hybrid semantic retrieval, constrained reasoning, and multilevel validation, within a unified, traceable workflow. This architecture ensures that LLM inference is guided by both linguistic context and formal knowledge from the LOINC hierarchy, enabling accurate and explainable mapping without reliance on language-specific rules or extensive manual annotation. We evaluate LabBridge on high-frequency, real-world laboratory datasets in Chinese and English, representing substantial cross-lingual and cross-institutional heterogeneity.

In summary, this study makes 3 key contributions: (1) we introduce LabBridge, a novel agentic framework that integrates LLMs with clinical ontologies for reliable laboratory standardization; (2) we provide empirical evidence of its superior performance across 2 languages (Chinese and English) and across model scales, highlighting the importance of system design over model size alone; and (3) we demonstrate a practical pathway toward scalable, auditable semantic interoperability in health care ecosystems, advancing the vision of globally connected health data.

## Methods

### Ethical Considerations

This study was approved by the Institutional Review Board of Guangdong Provincial People’s Hospital (approval number KY-N-2022-105-01). The requirement for informed consent was waived due to the retrospective use of deidentified data. The Guangdong Provincial People’s Hospital (GDPH) dataset consisted of anonymized clinical records. The MIMIC-IV-ED (Medical Information Mart for Intensive Care IV Emergency Department) database is a publicly available, deidentified dataset, accessed under credentialed certification (34034170) in compliance with its data use agreement. No compensation was provided because this was a retrospective secondary analysis of deidentified data. All data were handled in compliance with data protection regulations. No identifiable images were included in this manuscript.

### Problem Formulation and Core Challenges

**Figure 1.**
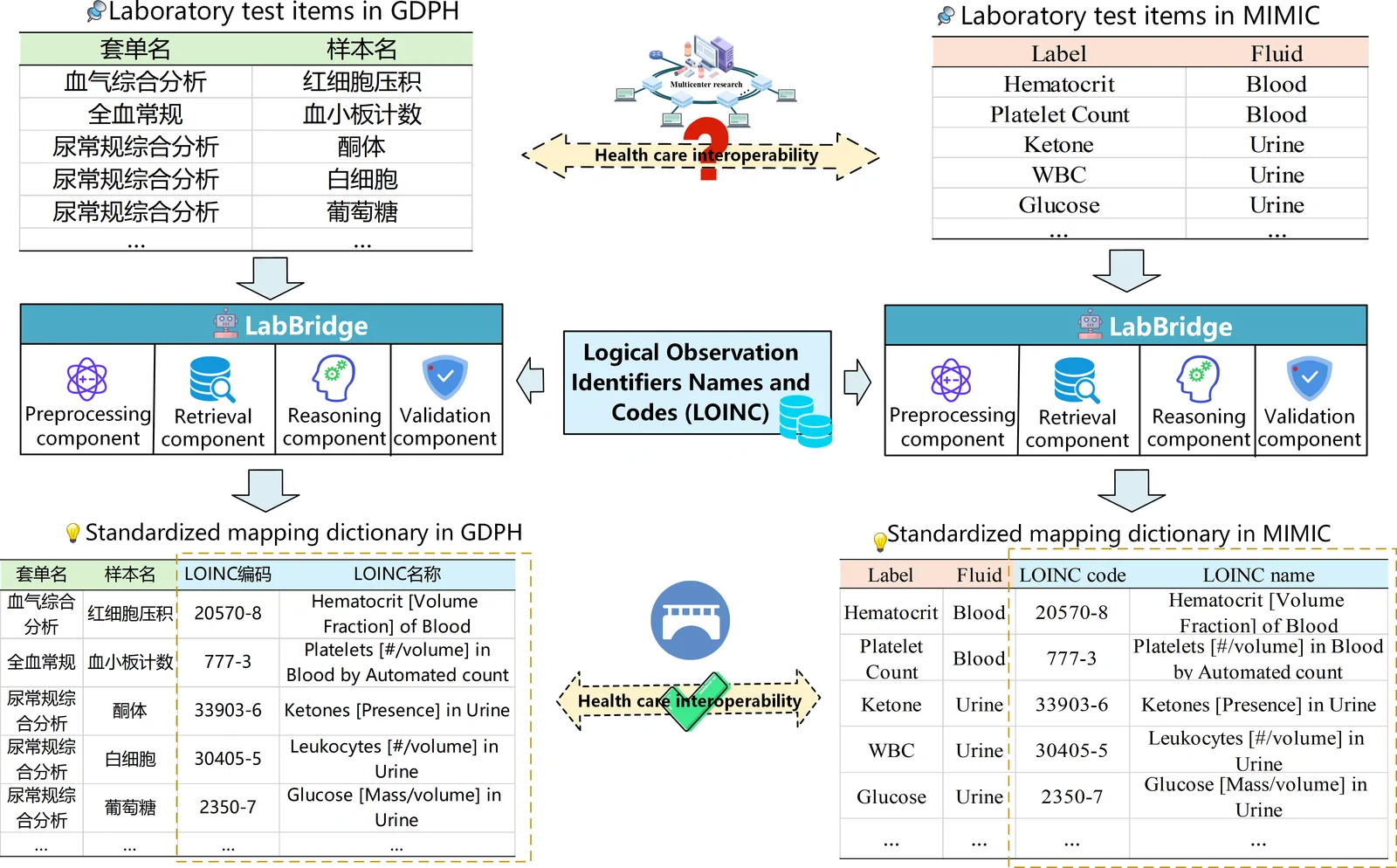
LabBridge enables cross-lingual and cross-institutional laboratory test standardization to LOINC, facilitating health care interoperability between Chinese (GDPH) and English (MIMIC) clinical systems. GDPH: Guangdong Provincial People’s Hospital; MIMIC: Medical Information Mart for Intensive Care.

The objective of this study is to learn an optimal mapping function f:T→C, which assigns the most semantically appropriate LOINC code $c^{\text{*}}\in C$ to each local laboratory test term t∈T. Here, $T=\{t_{1},t_{2},\ldots ,t_{n}\}$ represents a collection of textual descriptions from multiple languages (with Chinese and English as use cases in this study, eg, English “Glucose, blood” or Chinese “全血葡萄糖”), and $C=\{c_{1},c_{2},\ldots ,c_{m}\}$ denote the set of standardized LOINC codes (eg, 15074‐8, Glucose [Moles/volume] in blood). Given the scarcity of labeled multilingual training data, we frame this as a semantic similarity maximization problem (Figure 1):


(1) $$c^{\text{*}}\text{ = }\underset{c\in C}{argmax}\;similarity\left(t,c\right)$$

where *similarity* is quantified using cosine similarity between vector representations. Designing an effective and robust mapping function *f* requires addressing 3 inherent, nontrivial challenges: (1) multilingual variability, where identical clinical concepts are expressed with different lexical and syntactic structures across languages (eg, Chinese “血清葡萄糖测定” vs English “Glucose, serum”); (2) lexical heterogeneity, encompassing synonyms (eg, “HbA1c” vs “Glycated hemoglobin”), abbreviations (eg, “Na+” vs “Sodium”), and institution-specific naming conventions; and (3) contextual ambiguity, where terms such as “ACE” may refer to multiple clinical entities (eg, angiotensin-converting enzyme or acetylcholinesterase) depending on the test panel or clinical setting.

### LabBridge: An LLM-Based Agentic Framework

#### Architecture

**Figure 2.**
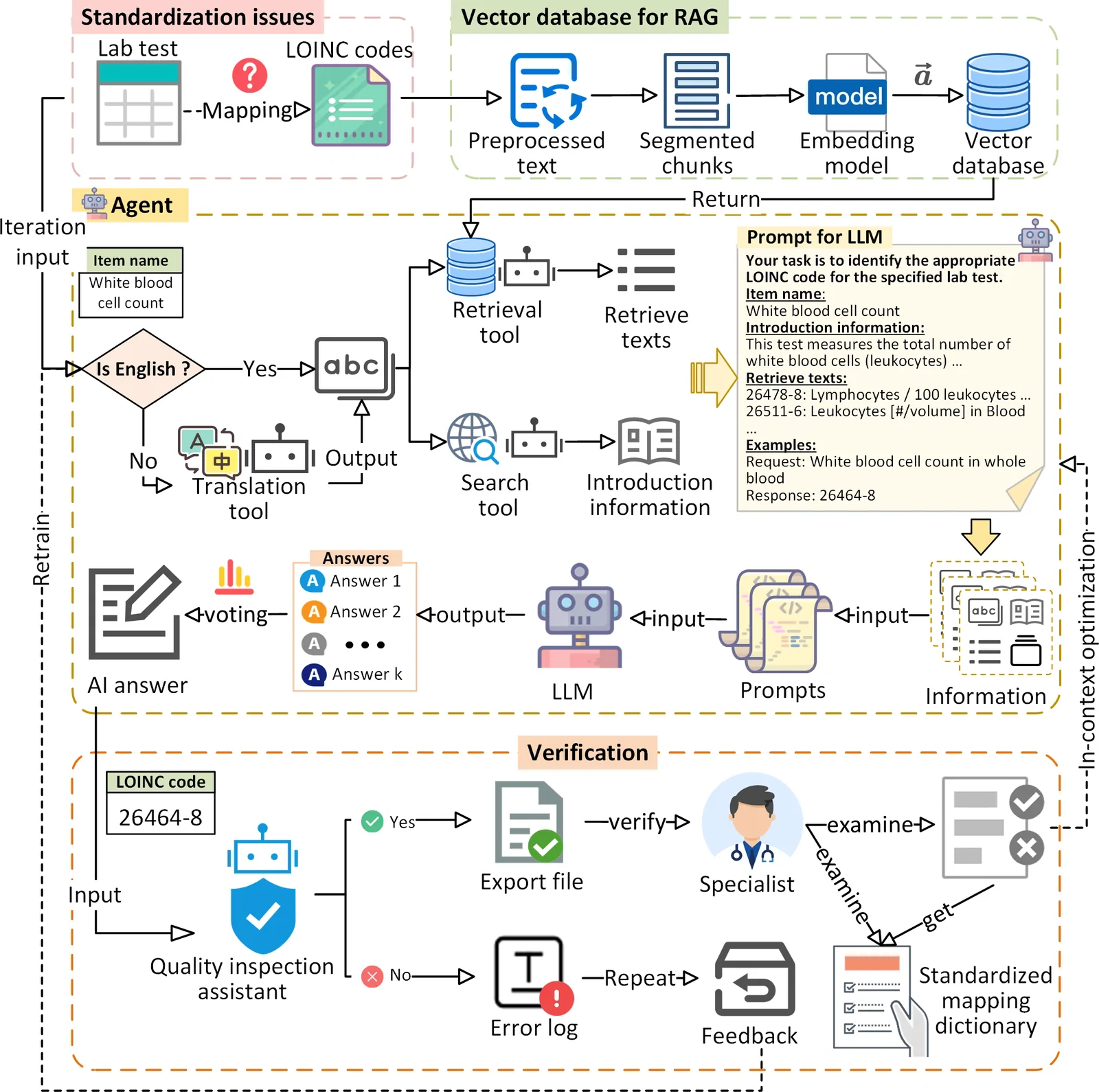
Integrated components of LabBridge include the following: language-aware preprocessing, hybrid retrieval, retrieval-augmented reasoning, and validation loop for Logical Observation Identifiers Names and Codes (LOINC) standardization mapping. LLM: large language model; RAG: retrieval-augmented generation.

LabBridge is architected as an LLM-based intelligent agentic framework that orchestrates end-to-end terminology standardization. Moving beyond static pipelines, it functions as a proactive coordinator that dynamically manages information flow and decision-making across 4 specialized, interoperable modules (Figure 2). This agentic design enables LabBridge to perceive raw input, plan and execute tool-augmented actions (preprocessing, retrieval, and reasoning), and refine outputs through iterative validation.

#### Language-Aware Preprocessing Module

Acting as the agent’s perceptual interface, this module first analyzes an input laboratory test term *t* to detect its language λ using a pretrained classifier. For any non-English term (λ ≠ *L*_English_), it invokes a clinical-domain translation tool to generate a normalized English representation *t*′, treating translation as a pragmatic normalization step rather than a perfect linguistic conversion:


(2) $$t^{\prime }=\mathrm{Translation}\;(t,\lambda \to L_{\text{English}})$$

To address cross-lingual variability, we employed the Baidu Translation API (medical domain) for domain-adaptive translation optimized for biomedical terminology. The API uses a signature-based authentication mechanism, ensuring stable performance across diverse datasets. This process transforms heterogeneous, multilingual inputs into a canonical English form suitable for downstream semantic processing.

#### Hybrid Retrieval Module

This module equips the agent with the ability to gather relevant evidence from a curated LOINC knowledge base (as detailed in the *Datasets and Reference Terminology* section). It employs 2 complementary tools operating in parallel:

- Vector retrieval tool: Official LOINC descriptions and related terms are encoded into dense vector representations using a multilingual embedding model. Specifically, we use OpenAI’s text-embedding-ada-002 model, which natively supports both English and Chinese, enabling cross-lingual semantic retrieval without separate monolingual models. The normalized query *t*′ is embedded as *v_t′_*, and a k-nearest neighbor search using cosine similarity returns the top-k semantically similar candidates *C_V_*(*t*′):

(3) $$v_{i}=\mathrm{Embedding}(s_{i}),C_{V}(t^{\prime })=\mathrm{kNN}\;\left(v_{t^{\prime }},\{v_{i}\}\right)$$

- Semantic search tool: To enhance semantic understanding, particularly for complex panel tests or rare terms, we implemented a background knowledge retrieval module integrating external search with LLM-based knowledge generation. This tool executes keyword matching and medical entity expansion using authoritative biomedical ontologies to retrieve the candidates. For external retrieval, the Bing Search API (Microsoft) was used to obtain real-time contextual information. Queries were formulated as “what is [item]” to retrieve concise and relevant web descriptions. Additionally, an LLM-based retrieval component using gpt-4o-mini (OpenAI GPT) generates short background explanations (approximately 60 words) focusing on the core medical semantics when external search results are insufficient or noisy.

The union *C_V_*(*t*′) ∪ *C_S_*(*t*′) forms a comprehensive, multiperspective evidence pool for the agent’s subsequent reasoning step. Notably, the semantic search tool can retrieve rich background information that complements the preprocessing pipeline, including abbreviation expansion (eg, “Hb” → “Hemoglobin”), unit and symbol standardization, and context-aware disambiguation (eg, differentiating “Ca” as calcium from “CA” as cancer antigen, or interpreting “CK” as creatine kinase rather than cytokeratin). These retrieved contextual cues help resolve ambiguities that cannot be addressed by translation alone.

#### Retrieval-Augmented Reasoning Module

This component serves as the core reasoning engine. It synthesizes gathered evidence by constructing a structured prompt (Figure S1 in Multimedia Appendix 1):


(4) $$Prompt_{dynamic}\text{ = }Concat\left(t^{\prime },C_{V}\left(t^{\prime }\right),C_{S}\left(t^{\prime }\right),E\right)$$

where *E* represents optional few-shot examples for in-context learning (ICL). The LLM, functioning as the agent’s reasoning kernel, generates a preliminary LOINC code prediction:


(5) $$l_{code}\text{ = }LLM\left(Prompt_{instr},Prompt_{dynamic};\theta \right)$$

Here, *θ* denotes the LLM parameters, and *Prompt*_instr_ is a fixed instruction template. To enhance decision stability and mitigate LLM stochasticity, the agent employs a majority-voting strategy over *K* =5 independent, parallel reasoning trials, selecting the final code *l*_output_ with the highest consensus.

#### Validation Loop Module

This module performs automated structural validation and supports iterative regeneration when invalid outputs are detected. Each candidate mapping undergoes an automated consistency check for format validity and ontological existence. If a prediction fails validation, a feedback-driven correction loop is initiated for up to *N* iterations. Structured error signals *e*^(^*^t^*^)^ (eg, “format violation” or “code not in ontology”) are generated and fed back to refine the reasoning module’s context *x*^(^*^t^*^)^, leading to a regenerated prediction *l*^(^*^t^*^+1)^:


(6) $$l^{\left(t\text{+}1\right)}\text{ = }f_{LLM}\left(x^{\left(t\right)}⊕e^{\left(t\right)}\right),t\text{ = }\left\{0,1,\ldots ,N\text{-}1\right\}$$

We set a maximum of *N*=5 optimization cycles per test item. If a valid LOINC mapping is not achieved after reaching this limit, the item is automatically logged as an unresolved case and flagged for expert review. This logging mechanism enables quantitative assessment of the system’s operational autonomy. Cases that persistently fail automated correction are exported as logs for expert confirmation. Rather than fine-tuning, we use ICL with task-specific prompts.

The task-specific prompts and in-context examples were developed during the framework development phase through iterative prompt engineering, repeated pilot testing, and domain expert review prior to formal evaluation. During this process, error signals from the validation loop were analyzed to identify recurring failure patterns and guide prompt refinement. Once finalized, the prompt configuration remained fixed across all evaluated models for the reported experiments. No online optimization occurred during evaluation. In future deployments, expert-validated pairs from the escalation workflow could be leveraged to further refine prompt design, enabling continuous performance improvement without model retraining.

### Datasets and Reference Terminology

To rigorously evaluate robustness across languages and institutions, we utilized 2 real-world laboratory datasets from emergency department (ED) patients (Table S1 in Multimedia Appendix 1): first, a Chinese dataset comprising over 5.85 million deidentified laboratory records from 7404 patients (2020‐2023) at GDPH. After deduplication based on test names, 317 high-frequency test items were retained for evaluation, where “high-frequency” was defined as test items used by more than 10% of patients. Second, an English dataset consisting of approximately 10.61 million laboratory records from 158,809 patients (2011‐2019) in the MIMIC-IV-ED database [23]. Following the same deduplication process, 288 high-frequency items were selected for evaluation, covering over 99.9% of all laboratory observations in the dataset.

The 2 datasets employed different filtering thresholds, which warrants clarification. The GDPH dataset was extracted from the complete hospital laboratory information system and thus contains a long tail of infrequently ordered tests (eg, allergen panels, hormone assays, and semen analyses), while the publicly released MIMIC-IV-ED database has undergone selective curation and predominantly includes routine blood and urine chemistries. To ensure a comparable evaluation baseline across the 2 datasets, retaining a sufficient number of high-frequency test items while maintaining clinical relevance, we applied a 10% patient-prevalence threshold for GDPH (given its full-spectrum composition) and an observation-coverage threshold (>99.9%) for MIMIC (Medical Information Mart for Intensive Care; to compensate for its selective composition and large cohort size). Although the numerical criteria differ, they are functionally equivalent: both were designed to isolate the core high-frequency test repertoire in each ED setting, ultimately yielding a comparable number of retained items (317 vs 288).

The reference laboratory terminology set was derived from the official LOINC Core List [24] (formally known as the “LOINC Universal Lab Order Codes Value Set,” version 2.78 used in the study). This laboratory value set, originally designed for order-entry system developers to deliver standard codes in health level 7 messages to laboratories, comprises the most frequently ordered laboratory tests. It was developed iteratively through both empirical and consensus-driven approaches. To ensure practical utility in our study context, we made minor refinements based on local clinical practice and naming conventions in Chinese hospital information systems, resulting in a final set of 1487 laboratory indicators most relevant to routine clinical care. The final mapping set was established by 2 trained graduate students in clinical medicine, who independently reviewed and annotated candidate codes using the official SearchLOINC web application. Interrater agreement between the 2 students, measured as percentage agreement, was 63.4%. Most disagreements arose from fine-grained specimen-type distinctions, for example, “serum“ vs “plasma” vs “whole blood,” or “arterial blood“ vs “venous blood” for blood gas analyses, reflecting the inherent ambiguity of the mapping task rather than low annotator reliability. In cases of disagreement or uncertainty, a deputy chief physician with extensive clinical and research experience made the final determination.

### Experimental Setup and Evaluation

To comprehensively evaluate the LabBridge framework, we established a rigorous experimental protocol encompassing baseline comparisons, model configuration analyses, and performance assessments across diverse conditions.

- Baseline methods: We benchmarked LabBridge against several representative approaches to contextualize its performance. These included (1) fuzzy string matching [25], implemented with both the FuzzyWuzzy and RapidFuzz libraries to assess lexical similarity; (2) vector-based matching using both BGE-M3 [26] and OpenAI’s text-embedding-ada-002 embeddings. For the Chinese dataset, translation-enhanced variants were additionally evaluated (BGE-M3+ translation and text-embedding-ada-002+ translation); (3) a conventional RAG pipeline without agentic coordination [16]; and (4) 2 LLM baselines: direct prompting (Direct-LLM, zero-shot) and ICL (few-shot) [21].
- Model configurations: To assess the generalizability and model-agnostic nature of our framework, LabBridge was integrated with 5 state-of-the-art LLMs, GPT-4o [27], GPT-4o-mini, Qwen-2.5-7B (Alibaba [28]), Llama-3.1-8B (Meta [29]), and DeepSeek-V3 [30], spanning diverse architectures, scales, and training corpora (Table S2 in Multimedia Appendix 1). Key hyperparameters, including retrieval depth (top-k), temperature, and few-shot example quantity, were systematically tuned for each model to optimize task-specific performance.
- Evaluation metric: The primary evaluation metric was mapping accuracy, defined as the proportion of local laboratory test names correctly linked to their corresponding LOINC codes. To thoroughly evaluate robustness at different frequency strata, performance was assessed across multiple frequency thresholds (Top@30%, 60%, 80%, and 100%), corresponding to test items ranked by their occurrence frequency in the respective datasets. In addition, pairwise model comparisons were conducted using the McNemar test under paired prediction settings. To control the false discovery rate due to multiple testing, the resulting *P* values were adjusted using the Benjamini-Hochberg procedure.
- Computational environment: All experiments were conducted on a high-performance computing server equipped with dual Intel Xeon Platinum 8462Y+processors, 2 TB of DDR5 RAM, and 2× NVIDIA A40 48 GB graphics processing units and running Ubuntu 22.04.4 LTS (Canonical Ltd). This configuration ensured efficient and reproducible execution of all inference and evaluation tasks.

## Results

### Overall Performance

Table 1 presents a comprehensive performance comparison of all evaluated methods, including fuzzy matching, vectorized matching, Direct-LLM, ICL, RAG, and LabBridge, for LOINC standardization across frequency-stratified subsets (Top@30% to Top@100%) for both the Chinese (GDPH) and English (MIMIC) datasets. LabBridge consistently achieves state-of-the-art performance across both language settings and all test frequency strata, significantly outperforming all baseline approaches. As shown in Table 1 (GDPH, Top@100%), LabBridge (with DeepSeek-V3) achieves the highest accuracy of 90%, outperforming the strongest vector-only baseline that uses the same embedding model (text-embedding-ada-002+ translation, 61%) by 29 percentage points and outperforming BGE-M3+ translation (50%) by 40 percentage points. Even with smaller open-weight models such as Qwen-2.5-7B and Llama-3.1-8B, LabBridge maintains high accuracy (80%‐84%), demonstrating its robustness to model choice. In the English MIMIC setting, where semantic matching is inherently less challenging, LabBridge still delivers superior or comparable results to RAG alone, achieving up to 90% accuracy (DeepSeek-V3 and GPT-4o-mini, Top@100%) and consistently outperforming Direct-LLM and ICL. Notably, conventional fuzzy matching methods (FuzzyWuzzy and RapidFuzz) and even standalone vectorized retrieval (BGE-M3) perform poorly on GDPH (<24%), highlighting the critical role of linguistic and ontological alignment in cross-lingual contexts.

**Table 1.** Performance comparison of different methods for Logical Observation Identifiers Names and Codes (LOINC) standardization across frequency-stratified subsets (Top@30%‐Top@100%) on the Chinese (GDPH) and English (MIMIC) datasets.

Methods	GDPH^a^				MIMIC^b^			
	Top@30%^c^	Top@60%^c^	Top@80%^c^	Top@100%^c^	Top@30%^c^	Top@60%^c^	Top@80%^c^	Top@100%^c^
Fuzzy matching								
FuzzyWuzzy	0.09	0.18	0.24	0.28	0.10	0.19	0.24	0.28
RapidFuzz	0.03	0.09	0.13	0.15	0.06	0.11	0.14	0.15
Vectorized matching								
BGE-M3	0.15	0.21	0.24	0.24	0.64	0.69	0.68	0.67
BGE-M3+ translation	0.45	0.49	0.49	0.50	—^d^	—^d^	—^d^	—^d^
Ada-002^e^	0.27	0.31	0.33	0.32	0.73	0.78	0.78	0.77
Ada-002^e^+ translation	0.53	0.59	0.62	0.61	—^d^	—^d^	—^d^	—^d^
Using Qwen-2.5-7B								
Direct-LLM^f^	0.00	0.00	0.00	0.00	0.00	0.00	0.00	0.00
ICL^g^	0.01	0.01	0.00	0.00	0.01	0.01	0.00	0.00
RAG^h^	0.39	0.43	0.45	0.42	0.79	0.77	0.76	0.77
LabBridge	0.82	0.82	0.80	0.83	0.81	0.82	0.82	0.81
Using Llama-3.1-8B								
Direct-LLM	0.00	0.00	0.00	0.00	0.00	0.00	0.00	0.00
ICL	0.01	0.01	0.00	0.00	0.01	0.01	0.00	0.00
RAG	0.24	0.30	0.31	0.29	0.64	0.66	0.65	0.63
LabBridge	0.80	0.84	0.84	0.83	0.80	0.81	0.82	0.82
Using GPT-4o-mini								
Direct-LLM	0.17	0.14	0.14	0.15	0.12	0.09	0.06	0.05
ICL	0.19	0.17	0.16	0.17	0.14	0.10	0.08	0.07
RAG	0.43	0.45	0.48	0.45	0.87	0.86	0.84	0.82
LabBridge	0.81	0.85	0.86	0.87	0.92**^i^**	0.89	0.86	0.90**^i^**
Using GPT-4o								
Direct-LLM	0.28	0.25	0.22	0.26	0.35	0.28	0.23	0.21
ICL	0.29	0.32	0.29	0.34	0.41	0.35	0.28	0.24
RAG	0.45	0.49	0.49	0.45	0.88	0.86	0.84	0.84
LabBridge	0.83	0.86	0.85	0.88	0.92**^i^**	0.89	0.87	0.89
Using DeepSeek-V3								
Direct-LLM	0.26	0.21	0.20	0.22	0.16	0.12	0.10	0.08
ICL	0.25	0.23	0.20	0.23	0.20	0.16	0.12	0.10
RAG	0.47	0.49	0.50	0.49	0.86	0.84	0.84	0.85
LabBridge	0.85^i^	0.89^i^	0.88^i^	0.90^i^	0.90	0.90^i^	0.88^i^	0.90^i^

a GDPH: Guangdong Provincial People’s Hospital.

b MIMIC: Medical Information Mart for Intensive Care.

c Top@num% refers to the top num% of laboratory test items ranked by frequency. All values represent exact-match accuracy.

d Not applicable, as translation is only performed for Chinese datasets and therefore not evaluated on English datasets.

e Ada-002 refers to OpenAI’s text-embedding-ada-002 model.

f LLM: large language model.

g ICL: in-context learning.

h RAG: retrieval-augmented generation.

i Values representing the highest accuracy for each dataset proportion.

To statistically validate the superiority of LabBridge over baseline methods, we performed the McNemar test to compare the pairwise classification outcomes of LabBridge against BGE-M3 and RAG on both datasets. As shown in Table S3 in Multimedia Appendix 1, all comparisons yielded statistically significant differences (*P*<.01) across all LLM backbones and both the GDPH and MIMIC datasets, confirming that LabBridge’s performance advantage is not due to random chance. Figure S2 in Multimedia Appendix 1 presents a stratified comparison of LabBridge (DeepSeek-V3) performance on high-frequency (Top@30%) vs low-frequency (Bottom@30%) test terms. For example, on the GDPH dataset, the results showed higher accuracy on low-frequency terms (93%) than on high-frequency terms (85%), indicating that mapping accuracy is determined more by semantic characteristics than by occurrence frequency alone. Representative examples in Figure S3 in Multimedia Appendix 1 illustrate this reliability: on the GDPH dataset, the query “全血常规中的红细胞比积对应的LOINC编码是多少?” (What is the LOINC code for Hematocrit in blood?) was correctly mapped to LOINC 20570‐8 (Hematocrit [Volume Fraction] of Blood); on the MIMIC dataset, “creatinine in blood” was accurately standardized to LOINC 38483‐4 (Creatinine [Mass/volume] in Blood). These cases exemplify LabBridge’s ability to resolve clinical intent across languages while preserving semantic and methodological fidelity.

### Sensitivity Analysis

**Figure 3.**
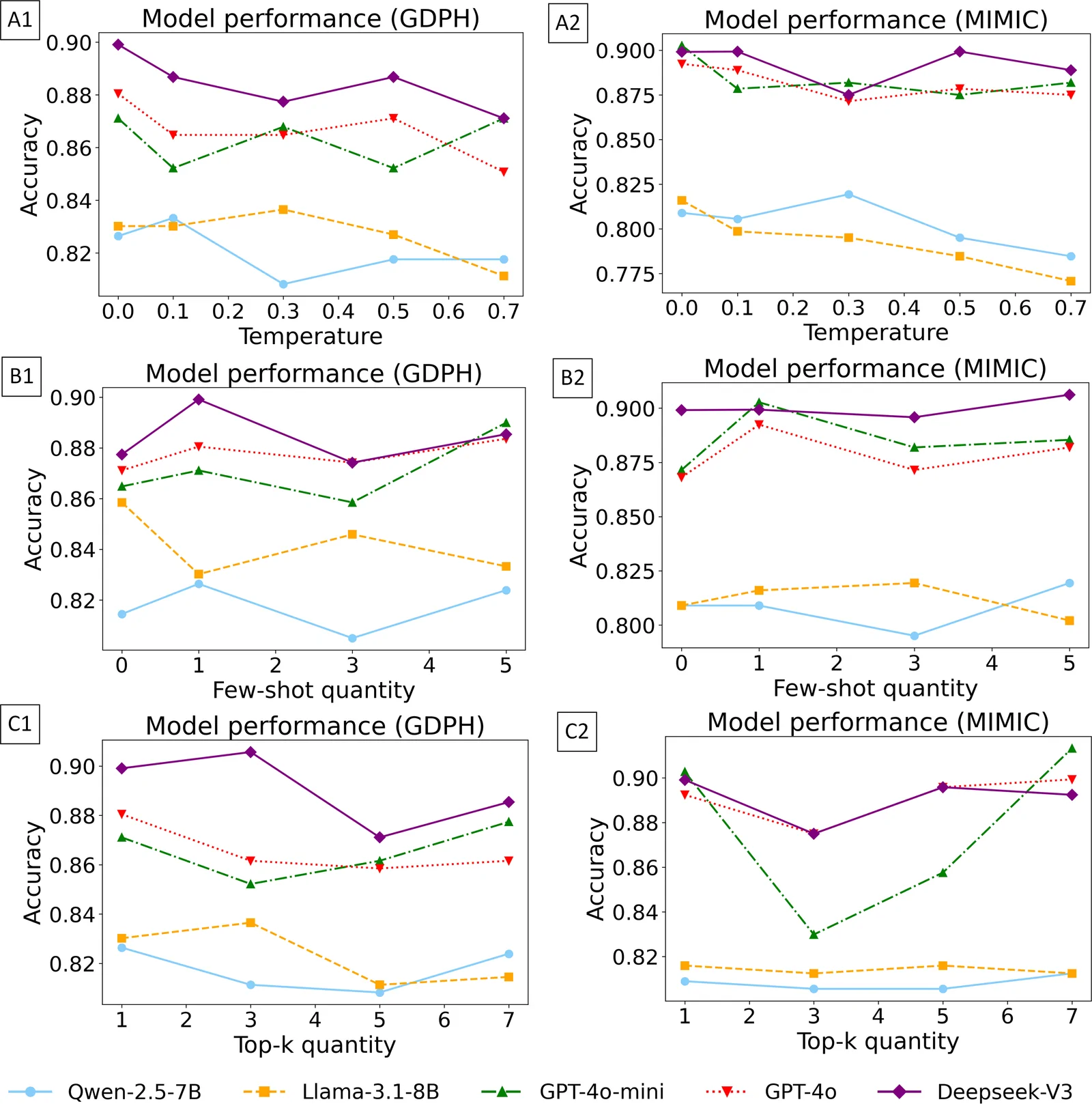
Sensitivity of LabBridge performance to hyperparameters: (A1-A2) temperature, (B1-B2) few-shot example count, and (C1-C2) top-k retrieval size, evaluated on the GDPH (Chinese) and MIMIC (English) datasets using 5 LLMs. GDPH: Guangdong Provincial People’s Hospital; MIMIC: Medical Information Mart for Intensive Care.

We conducted a comprehensive sensitivity analysis to assess how key hyperparameters, such as temperature, few-shot example counts, and top-k retrieval sizes, affect LabBridge’s performance across 5 LLMs in Chinese (GDPH) and English (MIMIC) settings (Figure 3). Model accuracy remains largely stable across temperatures from 0.0 to 0.7, with fluctuations within 2.5 percentage points for most models; GPT-4o-mini (MIMIC) and DeepSeek-V3 (GDPH) show exceptional consistency with fluctuations below 1.6 percentage points, whereas Qwen-2.5-7B and Llama-3.1-8B exhibit greater temperature sensitivity, especially on GDPH. The relationship between few-shot examples and performance is nonmonotonic and language-dependent: in GDPH, accuracy peaks at 1‐2 examples for GPT-4o-mini and DeepSeek-V3 before declining, likely due to prompt noise or overfitting, while in MIMIC, increasing examples up to 5 generally improves results, except for Llama-3.1-8B, which gains little. Similarly, optimal top-k values differ by model and language: GDPH benefits from moderate k (eg, k=3 for Llama-3.1-8B and DeepSeek-V3), whereas MIMIC performance plateaus at k=5‐7 for GPT models, while Qwen-2.5-7B and Llama-3.1-8B show minimal response to k, indicating a limited ability to utilize retrieved context. Overall, DeepSeek-V3 consistently achieves the highest performance across all hyperparameter settings, reaching peak accuracies of 90% on both GDPH and MIMIC. These findings demonstrate that LabBridge is inherently robust to moderate hyperparameter variations, particularly when paired with high-capacity LLMs, and that its performance can be further optimized through language-aware tuning of retrieval depth and prompt design.

To assess the impact of restricting the LOINC candidate space, we performed a supplementary sensitivity analysis using DeepSeek-V3. Starting from the curated Universal Lab Order Codes (1487 codes, yielding 90% accuracy on both datasets), we progressively expanded the candidate pool according to the official LOINC CLASS hierarchy, ultimately reaching the complete LOINC ontology (104,054 codes), as shown in Table S4 in Multimedia Appendix 1. Accuracy declined progressively with each expansion, falling to 41% (GDPH) and 39% (MIMIC) on the full ontology. These results confirm that using a constrained candidate set maintains high performance and avoids the accuracy degradation caused by massive, redundant LOINC alternatives. Moreover, across all candidate set sizes, our framework consistently outperformed direct LLM-based mapping methods (eg, 41% vs 22%, Table 1 and Table S4 in Multimedia Appendix 1), demonstrating its inherent robustness.

### Ablation Study

Table 2 presents the ablation study of LabBridge, showing the impact of removing key components (search, translation, retrieval, and verification) on LOINC standardization accuracy across models, languages, and test frequency strata. The search tool contributed modestly: its removal reduced accuracy by 1 to 5 percentage points on GDPH and 1 to 10 percentage points on MIMIC, with larger impacts observed on smaller models (eg, Qwen-2.5-7B) compared to larger ones (eg, DeepSeek-V3). The translation tool was critical for cross-lingual mapping on the Chinese dataset: its removal caused a severe 29 to 40 percentage point accuracy drops on GDPH across all models (eg, from 90% to 50% for DeepSeek-V3 at Top@100%). Note that the translation component was not ablated for the English MIMIC dataset, as the data are already in English. The retrieval tool proved indispensable for both datasets: removing it caused accuracy to drop to near zero (0.00‐0.36) across all models and frequency strata, indicating that the vector-based retrieval of LOINC candidates is a prerequisite for any subsequent reasoning. The verification module provided consistent but small gains: its removal led to 1 to 3 percentage-point declines across settings, with the largest drop (up to 3 percentage points) observed for Qwen-2.5-7B on MIMIC. Across all ablations, DeepSeek-V3 maintained the highest robustness, with accuracy remaining above 87% across most frequency tiers when translation and retrieval were retained. Notably, LabBridge demonstrated remarkable stability across test frequency strata, with accuracy varying only modestly from Top@30% to Top@100% across all configurations, confirming its generalizability to both common and less frequent laboratory tests.

**Table 2.** Ablation study of LabBridge showing the impact of removing key components (search, translation, retrieval, and verification) on Logical Observation Identifiers Names and Codes (LOINC) standardization accuracy, stratified by models, languages, and test frequency.

Methods	GDPH^a^				MIMIC^b^			
	Top@30%^c^	Top@60%^c^	Top@80%^c^	Top@100%^c^	Top@30%^c^	Top@60%^c^	Top@80%^c^	Top@100%^c^
Using Qwen-2.5-7B								
LabBridge	0.82	0.82	0.80	0.83	0.81	0.82	0.82	0.81
Without search tool	0.79	0.82	0.81	0.82	0.71	0.77	0.78	0.81
Without translate tool	0.47	0.48	0.49	0.46	—^d^	—^d^	—^d^	—^d^
Without retrieval tool	0.00	0.00	0.00	0.00	0.00	0.00	0.00	0.00
Without verification	0.80	0.82	0.81	0.80	0.78	0.80	0.79	0.80
Using Llama-3.1-8B								
LabBridge	0.80	0.84	0.84	0.83	0.80	0.81	0.82	0.82
Without search tool	0.75	0.81	0.80	0.82	0.75	0.77	0.76	0.77
Without translate tool	0.42	0.46	0.48	0.48	—^d^	—^d^	—^d^	—^d^
Without retrieval tool	0.00	0.00	0.00	0.00	0.00	0.00	0.00	0.00
Without verification	0.80	0.83	0.83	0.82	0.81	0.82	0.81	0.81
Using GPT-4o-mini								
LabBridge	0.81	0.85	0.86	0.87	0.92	0.89	0.86	0.90
Without search tool	0.81	0.85	0.86	0.86	0.90	0.86	0.85	0.88
Without translate tool	0.52	0.53	0.55	0.50	—^d^	—^d^	—^d^	—^d^
Without retrieval tool	0.13	0.13	0.13	0.14	0.15	0.09	0.06	0.05
Without verification	0.80	0.84	0.84	0.86	0.91	0.86	0.86	0.89
Using GPT-4o								
LabBridge	0.83	0.86	0.85	0.88	0.92	0.89	0.87	0.89
Without search tool	0.83	0.82	0.83	0.85	0.88	0.84	0.84	0.87
Without translate tool	0.51	0.52	0.54	0.51	—^d^	—^d^	—^d^	—^d^
Without retrieval tool	0.27	0.28	0.28	0.30	0.36	0.32	0.26	0.24
Without verification	0.81	0.84	0.84	0.85	0.90	0.89	0.86	0.88
Using DeepSeek-V3								
LabBridge	0.85	0.89	0.88	0.90	0.90	0.90	0.88	0.90
Without search tool	0.84	0.88	0.87	0.88	0.89	0.87	0.87	0.88
Without translate tool	0.52	0.53	0.55	0.50	—^d^	—^d^	—^d^	—^d^
Without retrieval tool	0.19	0.17	0.17	0.19	0.17	0.10	0.08	0.07
Without verification	0.83	0.87	0.86	0.88	0.90	0.89	0.88	0.90

a GDPH: Guangdong Provincial People’s Hospital.

b MIMIC: Medical Information Mart for Intensive Care.

c Top@num% refers to the top num% of laboratory test items ranked by frequency.

d Translation component ablation was not performed on the MIMIC dataset, as the original data are already in English.

### Error Analysis

To better understand the sources of mapping failures, we conducted an error analysis of all incorrect predictions. As shown in Figure S4 in Multimedia Appendix 1, LabBridge achieves 90% correct matches on both the Chinese (GDPH) and English (MIMIC) datasets. The remaining errors fall into 3 categories: mis-retrieval (4.4%‐5.0%), consistency errors (5.0% in both datasets), and translation errors (0.6% in GDPH). Of note, the translation module was designed to support non-English laboratory terms; in this study, it was applied exclusively to the Chinese GDPH dataset and was neither applied to nor evaluated on the English-native MIMIC dataset. Therefore, translation errors are absent in MIMIC. To assess error severity beyond binary accuracy, we categorized LabBridge mapping errors as exact matches, minor errors (eg, correct analyte with clinically similar specimen type such as whole blood vs plasma, or consistent clinical semantics despite minor technical variations), and major errors (eg, mismatched analyte, fundamentally different specimen type such as blood vs urine, or clinically unrelated mapping). As shown in Table S5 in Multimedia Appendix 1, the proportion of major errors was consistently lower in the top-performing models, particularly GPT-4o, GPT-4o-mini, and DeepSeek-V3, which achieved major error rates below 9% on the MIMIC dataset and below 12% on the GDPH dataset. A representative translation error case (corresponding to the translation error category in Figure S4 in Multimedia Appendix 1) is as follows: the Chinese term “尿液中粘液丝” (mucus threads in urine) was mistranslated as “Mucilaginous proteinuria,” leading the model to incorrectly retrieve 2888‐6 (Protein [Mass/volume] in Urine)—a major error, whereas the correct answer is 51478‐6 (Mucus [Number/volume] in Urine by Automated count). Furthermore, Table S6 in Multimedia Appendix 1 illustrates method differences using the hyaline casts query: traditional vector matching and direct LLM both yield major errors, while LabBridge achieves an exact match. In summary, although translation errors are rare, they can cause significant misalignment; the vast majority of mis-retrieval and consistency errors in both datasets are minor, indicating high overall clinical acceptability.

## Discussion

### Principal Findings

In this study, we introduced LabBridge, an agentic framework that reinterprets laboratory test standardization as a coordinated alignment process across linguistic, semantic, and ontological dimensions. Evaluated on Chinese and English real-world datasets, LabBridge achieved mapping accuracies of up to 90%, outperforming embedding-based (BGE-M3) and conventional RAG approaches, particularly on the Chinese dataset where linguistic variation is more fragmented. These results demonstrate that effective cross-lingual semantic interoperability can be achieved without extensive manual curation or language-specific engineering.

### Key Strengths and Clinical Implications

Our findings underscore 3 critical insights for clinical informatics. First, treating translation as pragmatic normalization, not as an end goal, enables robust cross-lingual alignment. By converting non-English terms to English solely to interface with LOINC’s canonical nomenclature, LabBridge sidesteps the pitfalls of imperfect machine translation while preserving clinical intent. This design proved especially effective for Chinese, where local naming conventions often diverge from official LOINC translations. Notably, non-English inputs in our current implementation rely on this dedicated translation component, as confirmed by the ablation results in Table 2. As LLMs continue to evolve, future work may incorporate multilingual capabilities directly, potentially reducing or eliminating this separate module. Nonetheless, the current design effectively demonstrates the feasibility of our agentic framework for cross-lingual semantic interoperability. Second, ontology-aware agent coordination matters more than model size alone. DeepSeek-V3, GPT-4o, and even smaller open-weight models (eg, Qwen-2.5-7B) performed comparably when embedded within LabBridge’s constrained reasoning pipeline, suggesting that system architecture can compensate for resource disparities, a crucial consideration for global health deployment. Third, the framework’s structured validation and feedback loop provide not only higher accuracy but also auditability: every mapping decision is traceable to retrieval evidence, consistency checks, and, when needed, expert correction. This transparency is essential for regulatory acceptance and clinical trust.

Interestingly, LabBridge performed better on low-frequency (Bottom@30%) than on high-frequency (Top@30%) test terms (93% vs 85% on the GDPH dataset; Figure S2 in Multimedia Appendix 1). This counterintuitive pattern, contrary to conventional data-driven models, is explained by 2 factors. First, LabBridge uses in-context optimization without fine-tuning, so all test concepts receive equal treatment regardless of frequency. Second, high-frequency tests often involve fine-grained distinctions (eg, “potassium” measured in serum, plasma, or whole blood), whereas low-frequency tests like “hepatitis B surface antibody” are more semantically distinctive. This finding further demonstrates that LabBridge maintains strong performance even on lower-frequency items within the evaluated datasets.

Regarding the reference set, although our primary evaluation was limited to the LOINC Core subset, the selected high-frequency test items represent the core set of routine laboratory tests in both ED datasets. Therefore, we believe the LONIC Core subset constraint does not undermine the validity of our findings. The performance gains were most pronounced in the Chinese dataset (+66 percentage points over BGE-M3), highlighting LabBridge’s value in low-resource, high-heterogeneity settings. In many non-English health care systems, standardized coding remains a bottleneck due to lexical diversity and limited natural language processing tooling. LabBridge offers a scalable pathway to bridge this gap, enabling institutions to leverage global terminologies like LOINC without abandoning local workflows. A key contribution of our framework is not to replace human expertise but to accelerate the clinical terminology mapping process. By automating candidate retrieval and ranking, this approach shifts the expert’s role from manual code lookup to candidate verification. Confirming a correct mapping is substantially faster than searching from scratch, thereby reducing time and cognitive burden even though final semantic correctness remains under expert oversight. This acceleration makes widespread terminology standardization clinically feasible.

### Comparison With Existing Approaches

Our work advances LOINC mapping beyond prior paradigms along 3 axes: robustness to linguistic diversity, integration of domain knowledge, and system-level adaptability. Traditional approaches, ranging from lexical matching to early machine learning on handcrafted features, fail to handle synonymy, multilingual variation, and contextual ambiguity [31]. While recent embedding-based and standard RAG methods improve semantic coverage, they remain brittle, sequential pipelines lacking mechanisms for error correction or ontological grounding [32,33]. LabBridge transcends these limitations through an agentic, feedback-driven architecture. Unlike static RAG, it implements a closed-loop process where validation outcomes iteratively refine retrieval and reasoning—mimicking expert-driven verification. Crucially, we decouple language normalization (via pragmatic translation) from semantic mapping, yielding a more scalable and auditable design than end-to-end cross-lingual models, whose opacity complicates error attribution. Ablation studies confirm this choice is pivotal: removing translation causes a 35% to 40% drop in accuracy on the Chinese dataset. Furthermore, rather than treating coding as zero-shot LLM generation, a practice prone to hallucination, LabBridge grounds inference in the LOINC ontology via hybrid retrieval and constrained decoding, ensuring output validity. The framework incorporates a feedback-driven validation loop together with carefully designed task-specific prompts and ICL, supporting reliable and auditable LOINC mapping without requiring large labeled datasets. This contrasts sharply with supervised fine-tuning or reinforcement learning [34], which remain impractical in low-resource, multilingual clinical settings. In summary, LabBridge demonstrates that ontology-aware orchestration of retrieval, reasoning, and validation offers a more feasible path to global semantic interoperability than data-intensive or black-box alternatives.

### Limitations

This study has several limitations. First, while our framework is designed to be multilingual, we only validated it on Chinese and English ED datasets; future research should expand to more languages and institutions, potentially exploring multilingual embedding spaces or language-specific LOINC proxies. Second, our evaluation metric relies on binary mapping accuracy, which fails to account for the clinical severity of errors; for instance, misclassifying a specimen type poses a greater risk than mapping to a parent code, so a weighted scoring system based on a 3-level severity classification (minor, moderate, major) would provide a more granular, clinically meaningful assessment. Third, our final verification focused on structural validity to mitigate LLM hallucinations, identifying candidate codes for expert review rather than providing a definitive mapping; while this significantly accelerates the manual curation process, future iterations should incorporate a human-in-the-loop mechanism to enable self-evolution and continuous model improvement. Fourth, to ensure mapping precision, we constrained the LOINC candidate set to a curated subset, which simplifies the retrieval task relative to the full ontology. Future work should explore scalable methods to relax this constraint. Fifth, the validation loop verifies only structural validity (code format and ontology existence), not semantic correctness. Therefore, it should not be interpreted as a clinically meaningful self-correction mechanism. Future work should incorporate semantic validation. Sixth, the raw interrater agreement of 63.4% meant that a single senior physician resolved 36.6% of disputes, which may introduce expert-specific bias into the gold standard. This moderate agreement partly reflects the inherent complexity of LOINC mapping, particularly fine-grained distinctions in specimen types or measurement contexts, which the senior physician adjudicated. The model’s 90% accuracy exceeding this human agreement reflects alignment with that senior physician’s reasoning rather than superior performance; multiexpert adjudication is needed in future work. Finally, although we compared multiple direct LLMs (including commercial ones) and demonstrated the robustness of our agent framework, it remains possible that the pretraining corpora of these LLMs may have contained the MIMIC-IV data dictionaries or LOINC mappings. Therefore, data contamination and memorization cannot be fully excluded as potential confounding factors.

### Future Directions and Broader Impact

Future research will extend LabBridge along several complementary trajectories. At the data layer, the framework requires validation on additional languages beyond Chinese and English, as well as multicenter datasets across diverse health care institutions, to fully assess its cross-lingual generalizability and real-world robustness. At the agent architecture level, future iterations will incorporate a human-in-the-loop memory mechanism. Specifically, the system will first query a memory bank of previously mapped terms; if a high-confidence match exists, it will return the result directly. Otherwise, it will invoke the full agentic framework to generate candidate mappings for expert verification, with successfully verified mappings subsequently added back to the memory bank. Over time, as the memory bank accumulates sufficient coverage, the frequency of full agent invocation and expert intervention is expected to decrease exponentially. To further refine mapping quality, future versions will introduce graded mapping confidence levels or quantitative scoring metrics to characterize the certainty of each mapping. The same framework architecture could also be extended to standardize other clinical domains (eg, radiology procedures to RadLex, medications to RxNorm [35]) and support dynamic updates as terminologies evolve. Finally, multicenter studies across different languages and health care systems will be essential to enhance global connectivity and interoperability, advancing the vision of seamless health data exchange worldwide.

### Conclusions

This study presents LabBridge, an LLM-based agentic framework that reinterprets laboratory test standardization as a coordinated alignment process across linguistic, semantic, and ontological dimensions. When evaluated on real-world Chinese and English ED datasets, this study demonstrates that LabBridge achieves up to 90% mapping accuracy, significantly outperforming baseline methods and transforming the expert workflow from manual LOINC lookup to efficient candidate verification. These findings confirm that effective cross-lingual semantic interoperability is achievable without extensive manual curation or language-specific engineering.

## Supplementary material

10.2196/92499 Multimedia Appendix 1 Development, evaluation, and validation of the LabBridge framework for laboratory test standardization.

## Abbreviations

ED: emergency department
GDPH: Guangdong Provincial People’s Hospital
ICL: in-context learning
LLM: large language model
LOINC: Logical Observation Identifiers Names and Codes
MIMIC: Medical Information Mart for Intensive Care
MIMIC-IV-ED: Medical Information Mart for Intensive Care IV Emergency Department
RAG: retrieval-augmented generation
