# Supplementary material for: Clinical Laboratory Terminology Standardization for Semantic Interoperability Using a Large Language Model–Based Agent: Methodological Study
Source: J Med Internet Res. 2026 Aug 20;28:e92499. doi: 10.2196/92499 (PMC13494520; doi:10.2196/92499)
Supplement: Supplementary file 1 [file jmir-v28-e92499-s001.docx]

**Clinical Laboratory Terminology Standardization for Semantic Interoperability Using a Large Language Model–Based Agent: Methodological Study**

**Authors:**

Lijuan Wu^1,2#^, PhD; Jinxin Huang^1#^, BS; Hongnian Wang^3#^, PhD; Liyi Mai^1^, BS; Xueyun Zhan^1^, MS; Xinrong He^1^, BS; Xiaotang Zhang^1^, MS; Huiying Liang^4^, PhD; Xin Li^5,6^, MD, PhD; Abdelouahab Bellou^1,5,6, 7, 8*^, MD, PhD.

**Multimedia Appendix 1**

**Figure S1.** Structure of the dynamic prompt template in the LabBridge framework, illustrating how retrieved evidence and instructions are integrated for laboratory test standardization.

**Figure S2.** Performance comparison of LabBridge (using DeepSeek-V3) on high-frequency (Top@30%) versus low-frequency (Bottom@30%) laboratory test terms.

**Figure S3.** Case study of standardization performance on representative GDPH and MIMIC examples, demonstrating effectiveness in multilingual and domain-adaptive scenarios.

**Figure S4.** Distribution of error types in LabBridge’s LOINC mapping: (A) Chinese (GDPH) and (B) English (MIMIC) datasets. Examples illustrate representative cases of translation, consistency, and mis-retrieval errors.

**Table S1.** Summary statistics and characteristics of the GDPH and MIMIC laboratory datasets.

**Table S2.** Summary of large language models and configurations used in the experiments.

**Table S3.** Statistical comparison between LabBridge and baseline methods (BGE-M3 and RAG) using McNemar’s test on the Chinese (GDPH) and English (MIMIC) datasets.

**Table S4.** Sensitivity analysis with progressively expanded candidate spaces using DeepSeek-V3.

**Table S5.** Severity-aware evaluation of LOINC mapping performance across models and datasets.

**Table S6.** Comparison of different methods using a representative example.

**Figure S1.** Structure of the dynamic prompt template in the LabBridge framework, illustrating how retrieved evidence and instructions are integrated for laboratory test standardization.

**Figure S2.** Performance comparison of LabBridge (using DeepSeek-V3) on high-frequency (Top@30%) versus low-frequency (Bottom@30%) laboratory test terms.

**Figure S3.** Case study of standardization performance on representative GDPH and MIMIC examples, demonstrating effectiveness in multilingual and domain-adaptive scenarios.

**Figure S4.** Distribution of error types in LabBridge’s LOINC mapping: (A) Chinese (GDPH) and (B) English (MIMIC) datasets. Examples illustrate representative cases of translation, consistency, and mis-retrieval errors.

**Table S1.** Summary statistics and characteristics of the GDPH and MIMIC laboratory datasets.

| **Attribute** | **GDPH** | **MIMIC** |
| --- | --- | --- |
| Source Institution | Guangdong Provincial People’s Hospital in China | Beth Israel Deaconess Medical Center in America |
| Collection Period | 2020 –2023 | 2011 – 2019 |
| Patient Population | Emergency Department | Emergency Department |
| Total Records | 5,854,433 | 10,613,341 |
| Selected for Evaluation | 317 | 288 |
| Specimen Type Included | Yes | Yes |
| Language | Chinese | English |

**Table S2.** Summary of large language models and configurations used in the experiments.

| **Models** | **Qwen-2.5-7B** | **Llama-3.1-8B** | **GPT-4o-mini** | **GPT-4o** | **DeepSeek-V3** |
| --- | --- | --- | --- | --- | --- |
| Model version | Qwen2.5-7B-Instruct | Llama-3.1-8B-Instruct | GPT-4o-mini | GPT-4o | DeepSeek-V3 |
| Parameter size | 7B | 8B | Not disclosed | Not disclosed | 671B |
| Open/Closed source | Open-source | Open-source | Closed-source | Closed-source | Open-source |
| Max context length | 128K | 128K | 128K | 128K | 128K |
| GPU Memory requirement | $\text{≥}$16GB | $\text{≥}$16GB | Not disclosed | Not disclosed | $\text{≥}$380GB |
| Inference environment | Local | Local | Cloud API | Cloud API | Cloud API |
| Release date | 2024-09-18 | 2024-07-23 | 2024-07-18 | 2024-08-06 | 2024-12-26 |

**Table S3.** Statistical comparison between LabBridge and baseline methods (BGE-M3 and RAG) using McNemar’s test on the Chinese (GDPH) and English (MIMIC) datasets.

| **LabBridge Model** | **GDPH** | | | | |  | **MIMIC** | | | | |
| --- | --- | --- | --- | --- | --- | --- | --- | --- | --- | --- | --- |
|  | BGE‑M3 | |  | RAG | |  | BGE‑M3 | |  | RAG | |
|  | χ² | *P* |  | χ² | *P* |  | χ² | *P* |  | χ² | *P* |
| Qwen2.5 | 167.51 | P<.001 |  | 100.81 | P<.001 |  | 26.30 | P<.001 |  | 8.02 | P=.005 |
| Llama3.1 | 168.50 | P<.001 |  | 146.61 | P<.001 |  | 13.29 | P<.001 |  | 16.01 | P<.001 |
| GPT-4o-mini | 180.36 | P<.001 |  | 105.06 | P<.001 |  | 44.18 | P<.001 |  | 10.26 | P=.001 |
| GPT-4o | 172.41 | P<.001 |  | 93.13 | P<.001 |  | 48.94 | P<.001 |  | 9.50 | P=.002 |
| DeepSeek-V3 | 186.33 | P<.001 |  | 117.31 | P<.001 |  | 48.19 | P<.001 |  | 9.50 | P=.002 |
| **Note:** Raw P values are from McNemar’s test. To account for multiple testing (20 comparisons), the Benjamini‑Hochberg (BH) procedure was applied with a false discovery rate (FDR) of 0.05. After BH correction, all comparisons remain statistically significant; the largest adjusted P value among the 20 tests was 0.005. Therefore, correcting for multiple testing does not change any conclusion. | | | | | | | | | | | |

**Table S4.** Sensitivity analysis with progressively expanded candidate spaces using DeepSeek-V3.

| **Candidate Space** | **Size** | **GDPH Accuracy** | **MIMIC Accuracy** |
| --- | --- | --- | --- |
| Universal Lab Order Codes ^a^ | 1,487 | 0.90 | 0.90 |
| Expanded Space I: Universal Codes + Chemistry and Routine Testing ^b^ | 21020 | 0.58 | 0.66 |
| Expanded Space II: Space I + Hematology and Coagulation ^b^ | 24209 | 0.44 | 0.45 |
| Expanded Space III: Space II + Microbiology and Infection ^b^ | 40551 | 0.46 | 0.44 |
| Expanded Space IV: Space III + Immunology and Cellular Markers ^b^ | 42729 | 0.46 | 0.43 |
| Full Laboratory LOINC Space ^c^ | 49148 | 0.42 | 0.41 |
| Full LOINC Ontology ^c^ | 104054 | 0.41 | 0.39 |
| ^a^ Universal Lab Order Codes denotes the curated candidate set used by LabBridge.  ^b^Expanded candidate spaces were constructed by progressively incorporating additional laboratory concepts based on the official LOINC CLASS hierarchy. Specifically, concepts belonging to the CHEM, DRUG/TOX, and UA classes were added as the Chemistry and Routine Testing domain; HEM/BC, COAG, and BLDBK as the Hematology and Coagulation domain; MICRO, ABXBACT, and SERO as the Microbiology and Infection domain; and HLA and CELLMARK as the Immunology and Cellular Markers domain.  ^c^ Full-space baselines include the complete laboratory subset of LOINC (49,148 laboratory concepts) and the entire LOINC ontology (104,054 concepts), representing the largest candidate spaces evaluated. | | | |

**Table S5.** Severity-aware evaluation of LOINC mapping performance across models and datasets.

| **LabBridge Model** | **Dataset** | **Total Num** | **Exact**  **n (%) ^a^** | **Minor ^b^**  **n (%)** | **Major ^c^**  **n (%)** | **Weighted Acc ^d^** | |
| --- | --- | --- | --- | --- | --- | --- | --- |
| Qwen2.5 | GDPH | 317 | 264 (83.3%) | 4 (1.3%) | 49 (15.5%) | 84.0% | |
|  | MIMIC | 288 | 234 (81.3%) | 5 (1.7%) | 49 (17.0%) | 82.2% | |
| Llama3.1 | GDPH | 317 | 262 (82.6%) | 7 (2.2%) | 48 (15.1%) | 83.7% | |
|  | MIMIC | 288 | 236 (81.9%) | 6 (2.1%) | 46 (16.0%) | 83.0% | |
| GPT-4o-mini | GDPH | 317 | 275 (86.8%) | 5 (1.6%) | 37 (11.7%) | 87.6% | |
|  | MIMIC | 288 | 258 (89.6%) | 6 (2.1%) | 24 (8.3%) | 90.7% | |
| GPT-4o | GDPH | 317 | 278 (87.7%) | 5 (1.6%) | 34 (10.7%) | 88.5% | |
|  | MIMIC | 288 | 257 (89.2%) | 8 (2.8%) | 23 (8.0%) | 90.6% | |
| DeepSeek-V3 | GDPH | 317 | 285 (89.9%) | 6 (1.9%) | 26 (8.2%) | 90.9% | |
|  | MIMIC | 288 | 258 (89.6%) | 9 (3.1%) | 21 (7.3%) | 91.2% | |
| ^a^ Values are reported as percentages of the total test set. Percentages may not sum to 100 because of rounding.  ^b^ minor errors (e.g., correct analyte with clinically similar specimen type such as whole blood vs. plasma, or consistent clinical semantics despite minor technical variations)  ^c^ major errors (e.g., mismatched analyte, fundamentally different specimen type such as blood vs. urine, or clinically unrelated mapping)  ^d^ Weighted accuracy is computed as Exact + 0.5 × Minor. | | | | | | |  |

**Table S6.** Comparison of different methods using a representative example.

| **Method** | **Query** | **Retrieved Result** | **Interpretation & Issues** |
| --- | --- | --- | --- |
| **Vectorized matching** ^a^ | What is the LOINC code for Hyaline Casts in Urine? | 53284-6 (Broad casts [#/volume] in Urine by Computer assisted method) | **Incorrect:** Retrieved a different cast subtype (“Broad” instead of “Hyaline”); semantic matching failure. |
| **Directly LLM**  **(No RAG)** ^b^ | What is the LOINC code for Hyaline Casts in Urine? | 5804-4 (Hyaline casts [Presence] in Urine by Microscopy) | **Incorrect:** Hallucinated result; the model generated a non-existent LOINC code (5804-4) and mistakenly chose an absence code. |
| **RAG-based LLM** ^b^ | What is the LOINC code for Hyaline Casts in Urine? | **Multiple:** 53284-6 (Broad casts [#/volume] in Urine by Computer assisted method), 105091-3 (Hyaline casts [#/volume] in Urine) | **Partially Correct:** Includes correct code but lacks disambiguation; risk of choosing incorrect cast type or method without context. |
| **LabBridge** ^b^ | What is the LOINC code for Hyaline Casts in Urine? | 105091-3 (Hyaline casts [#/volume] in Urine) | **Correct:** Accurately resolves the cast subtype, measurement unit, and method through context understanding and code verification. |
| ^a^ The traditional vectorized method uses the BGE-M3 model.  ^b^ LLM-based methods (i.e., Direct LLM, RAG-based, and LabBridge) are implemented using DeepSeek-V3. | | | |
